# Supplementary material for: Increased occurrences of consecutive La Niña events under global warming
Source: Nature. 2023 Jul 26;619(7971):774–81. doi: 10.1038/s41586-023-06236-9 (PMC10371868; doi:10.1038/s41586-023-06236-9)
Supplement: Supplementary file 1 — This file contains Supplementary Figs. 1–13 and Supplementary Table 1 that support the results presented. [file 41586_2023_6236_MOESM1_ESM.pdf]

nature portfolio

https://doi.org/10.1038/s41586-023-06236-9

Supplementary information

Increased occurrences of consecutive La Niña events under global warming

In the format provided by the
authors and unedited

Supplementary Information *for*

Increased occurrences of consecutive La Niña events under global warming

Tao Geng[1,2], Fan Jia[3,1*], Wenju Cai[2,4,5*], Lixin Wu[1,2], Bolan Gan[2,1], Zhao Jing[2,1], Shujun Li[2,1], and Michael J. McPhaden[6].

[1]Laoshan Laboratory, Qingdao, China.

[2]Frontiers Science Center for Deep Ocean Multispheres and Earth System (FDOMES) and Physical Oceanography Laboratory, Ocean University of China, Qingdao, China.

[3]CAS Key Laboratory of Ocean Circulation and Waves, Institute of Oceanology, Chinese Academy of Sciences, Qingdao, China.

[4]Centre for Southern Hemisphere Oceans Research (CSHOR), CSIRO Oceans and Atmosphere, Hobart, Australia.

[5]State Key Laboratory of Loess and Quaternary Geology, Institute of Earth Environment, Chinese Academy of Sciences, Xi'an, China.

[6]NOAA/Pacific Marine Environmental Laboratory, Seattle, Washington, USA.

*Correspondence to: Fan Jia (jiafan@qdio.ac.cn) and Wenju Cai (wenju.cai@csiro.au)

This file includes:

Supplementary Figs. 1 to 13
Supplementary Table 1

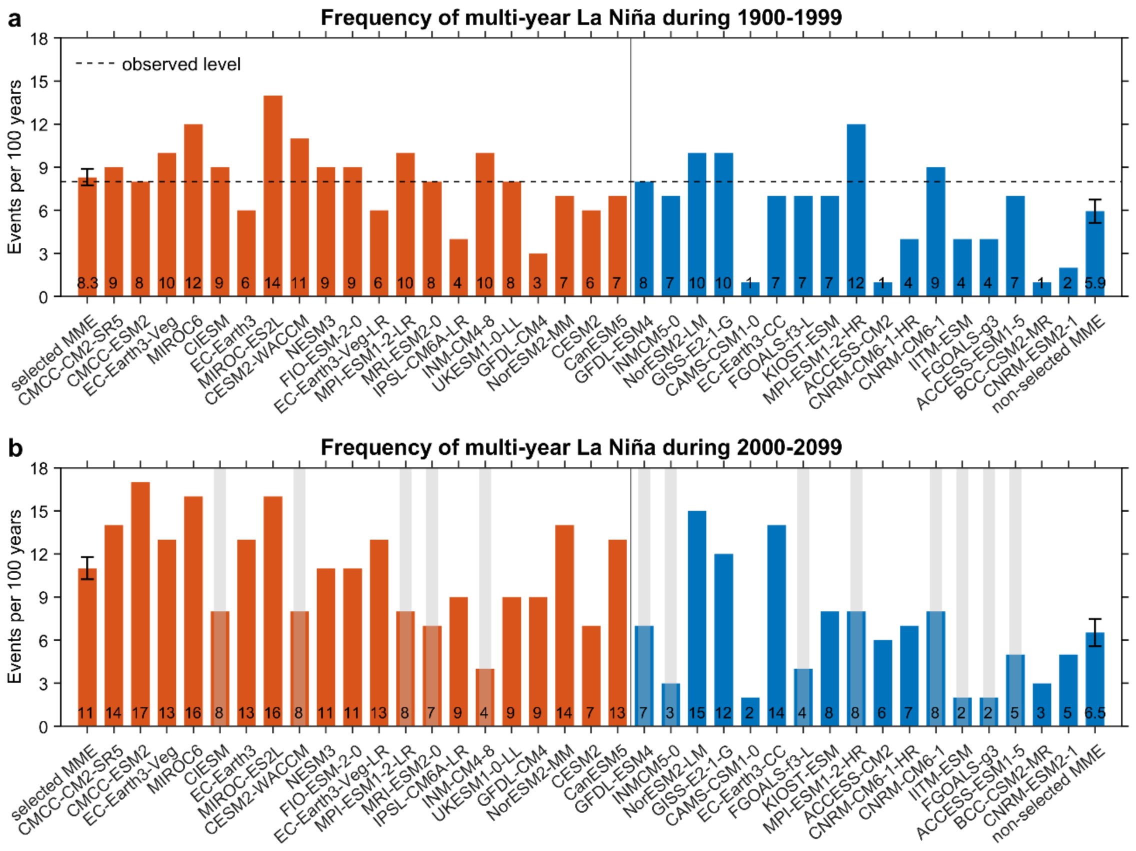

Supplementary Fig. 1 | Frequency of multi-year La Niña events in individual models. As in Fig.1a, but for multi-year La Niña frequency (events per 100 years, labeled on respective bars) over (**a**) 1900-1999 and (**b**) 2000-2099 under SSP585 in each of the 37 CMIP6 models. The vertical line separates selected models (orange bars) from non-selected models (blue bars). The error bar denotes 1.0 standard deviation (s.d.) of the inter-model spread in the selected (non-selected) multi-model ensemble (MME). The horizontal dashed line in **a** indicates observation. Models that simulate a decreased frequency in 2000-2099 are greyed out in **b**.

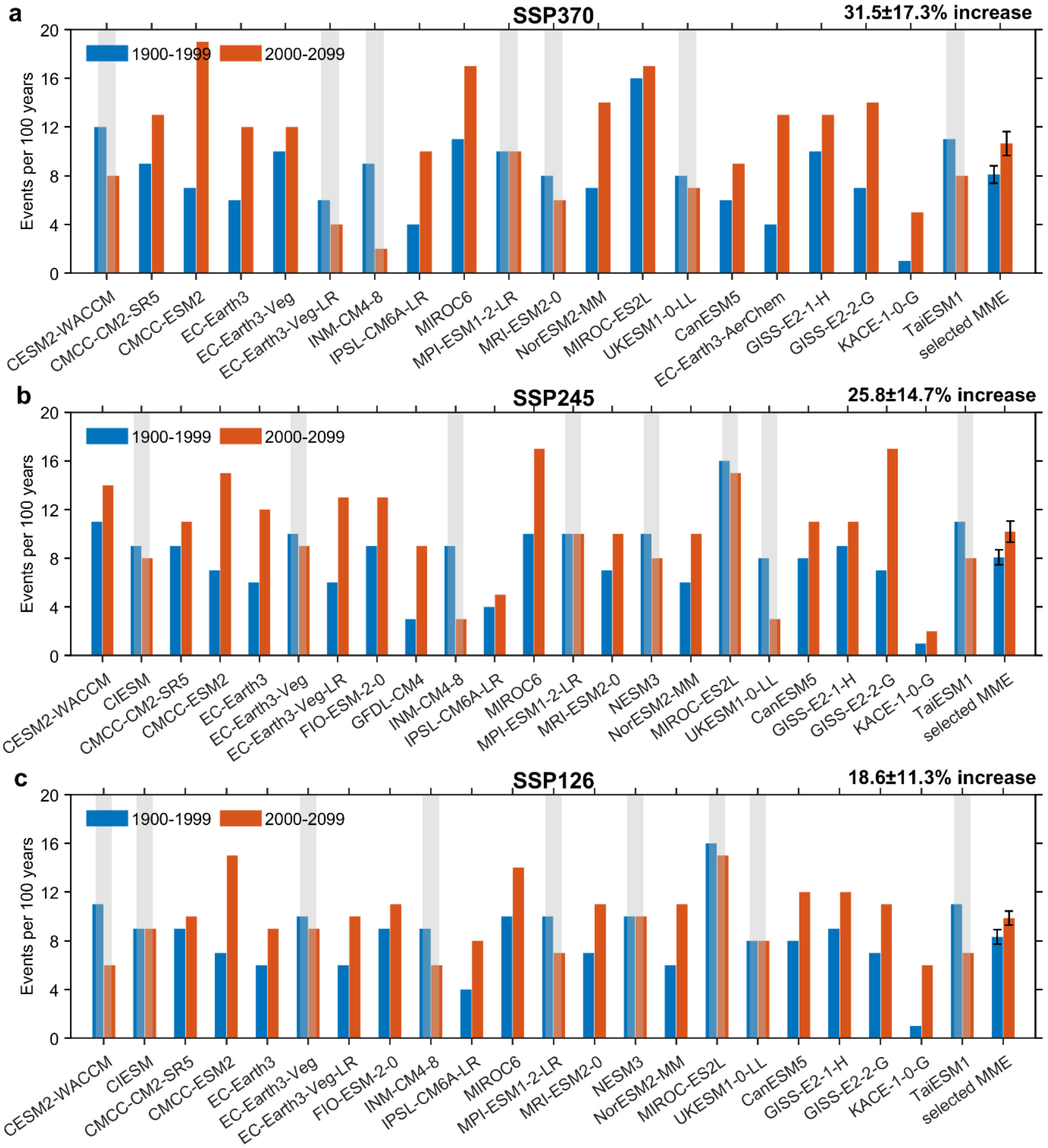

Supplementary Fig. 2 | Sensitivity of projected increase in multi-year La Niña frequency to emission scenarios. As in Fig. 2a, but for a comparison of multi-year La Niña frequency (events per 100 years) over 1900-1999 (blue bars) and 2000-2099 (red bars) under (**a**) SSP370, (**b**) SSP245 and (**c**) SSP126 emission scenarios. Models that do not simulate an increase are greyed out. Error bars on the multi-model ensemble mean (MME) are calculated as 1.0 s.d. of 10,000 inter-realizations of a Bootstrap method. All available models after selection (based on a positive Niño3.4 SST skewness) are used for each scenario. The MME increase is indicated in the top right. In all emission scenarios, a statistically significant increase in multi-model mean frequency is generated, and the inter-model consensus increases with the intensity of greenhouse gas emissions.

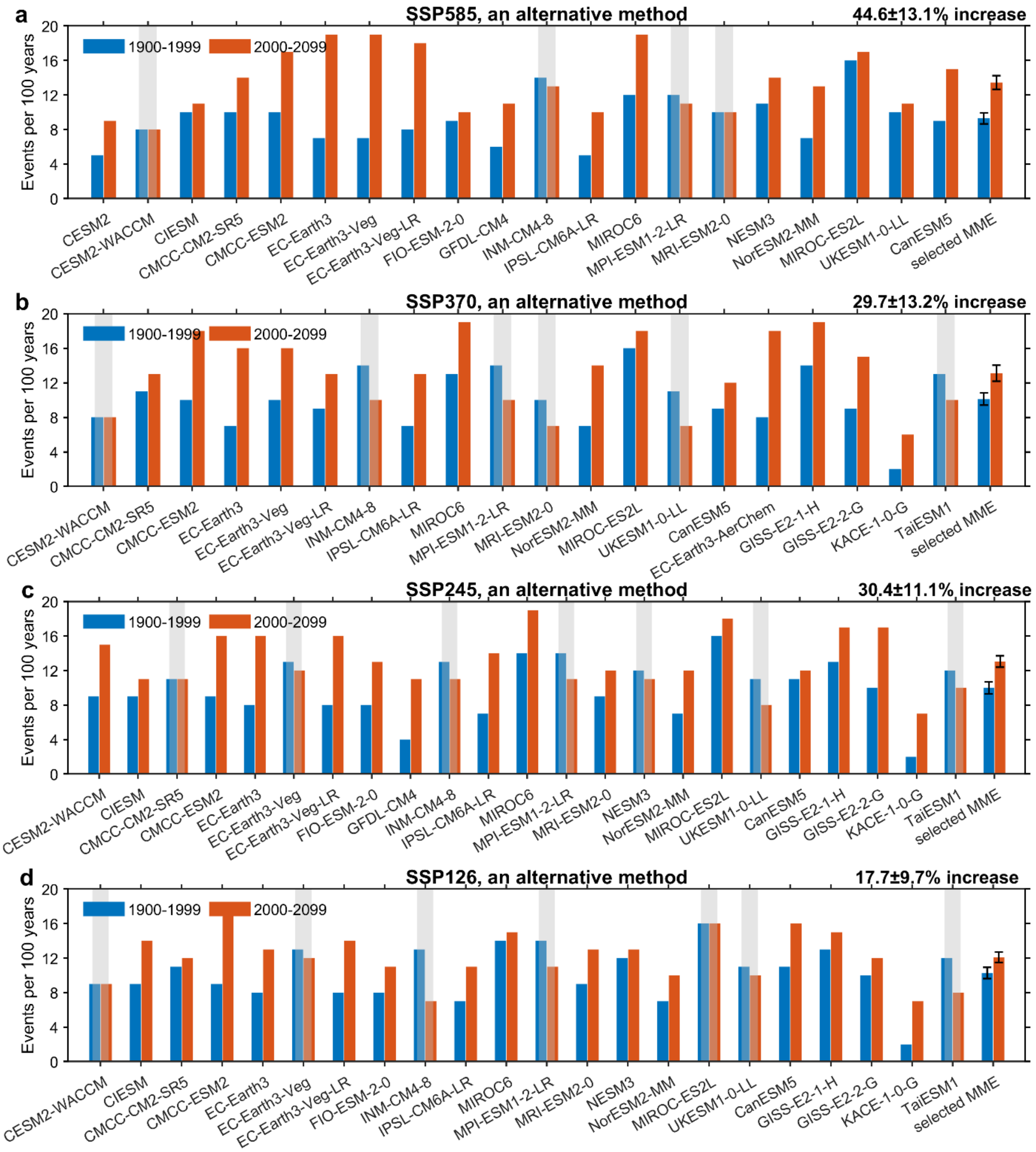

Supplementary Fig. 3 | Sensitivity of projected increase in multi-year La Niña frequency to definition. As in Fig. 2a, but for a comparison of multi-year La Niña frequency (events per 100 years) over 1900-1999 (blue bars) and 2000-2099 (red bars) using an alternative method, in which a multi-year La Niña event is defined as when the Niño3.4 index falls below -0.75 standard deviations in any month during October (0) to February (1) and remains below -0.5 standard deviations in any month during October (1) to February (2) under (**a**) SSP585, (**b**) SSP370, (**c**) SSP245 and (**d**) SSP126 emission scenarios. Models that do not simulate an increase are greyed out. Error bars on the multi-model ensemble mean (MME) are calculated as 1.0 s.d. of 10,000 inter-realizations of a Bootstrap method. The MME increase are indicated in the top right. An increased frequency of multi-year La Niña is insensitive to varying definitions.

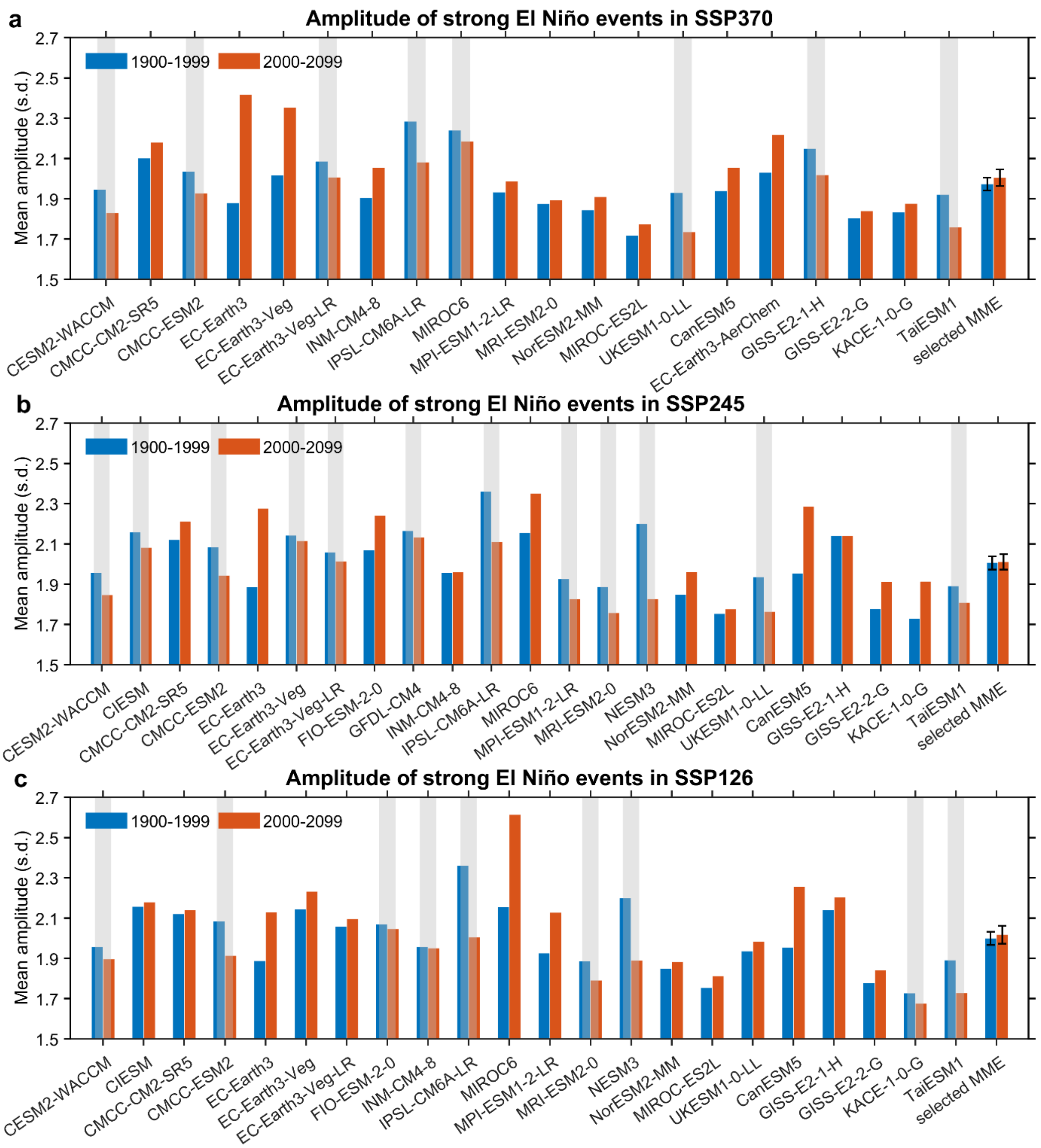

Supplementary Fig. 4 | No impact from changing amplitude of strong El Niño in other emission scenarios. As in Extended Data Fig.5a, but for a comparison of mean amplitude (s.d.) of strong El Niño events over 1900-1999 (blue bars) and 2000-2099 (red bars) in the selected models under (**a**) SSP370, (**b**) SSP245, and (**c**) SSP126 scenarios. Models that simulate a decrease are greyed out. Error bars on the multi-model ensemble mean (MME) are calculated as 1.0 standard deviation of 10,000 inter-realizations of a Bootstrap method.

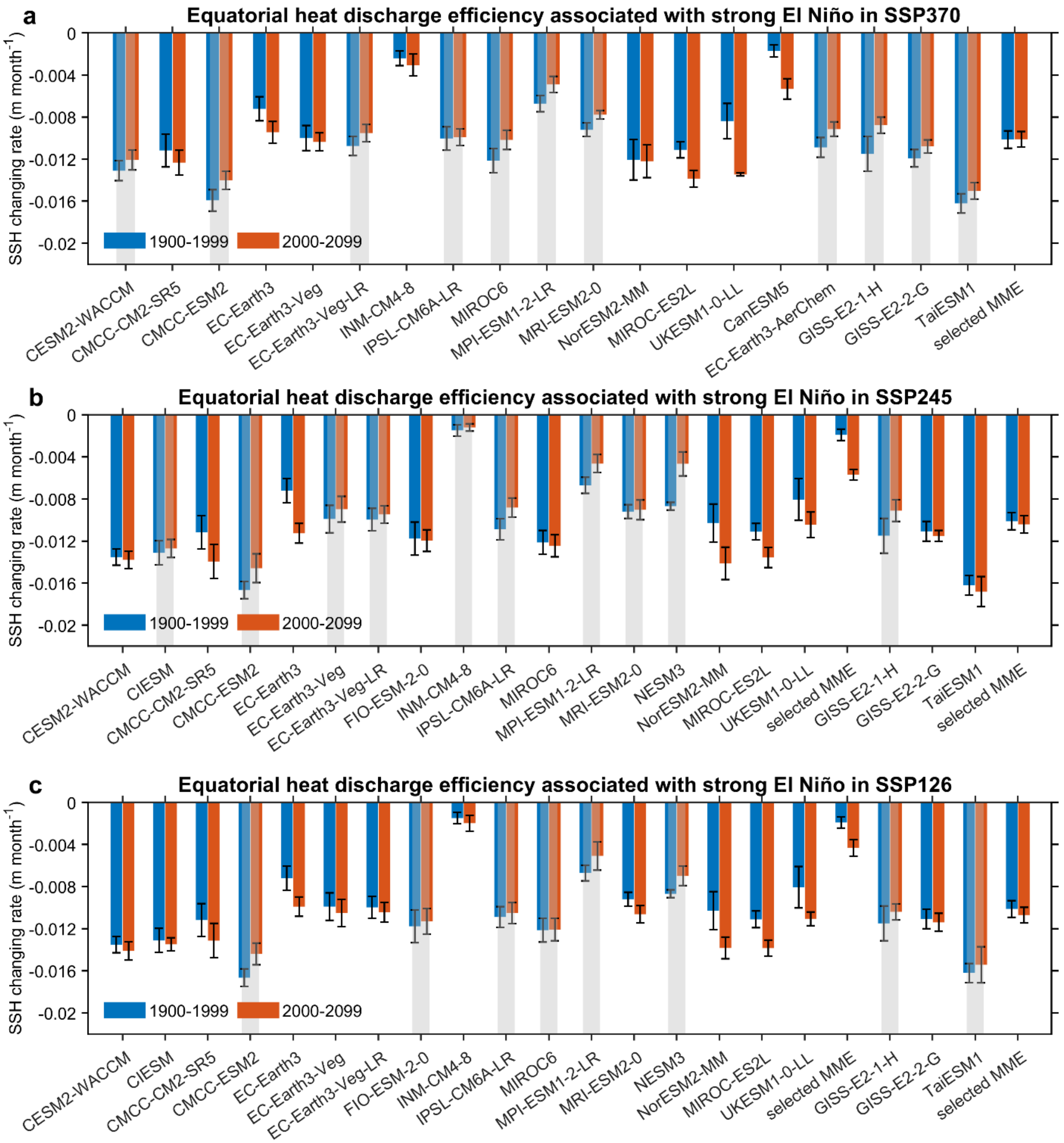

Supplementary Fig. 5 | No impact from changing equatorial heat discharge rate associated with strong El Niño in other emission scenarios. As in Extended Data Fig. 6b, but for a comparison of mean DJFMAM changing rate of SSH (m month^{-1}) during strong El Niño years, which measures the equatorial heat discharge rate associated with strong El Niño events, over 1900-1999 (blue bars) and 2000-2099 (red bars) in the selected models under (**a**) SSP370, (**b**) SSP245, and (**c**) SSP 126 scenarios. Models that simulate a decrease are greyed out. Error bars denote 1.0 s.d. of 10,000 inter-realizations using a Bootstrap method of the samples for each model simulation and the multi-model ensemble mean (MME).

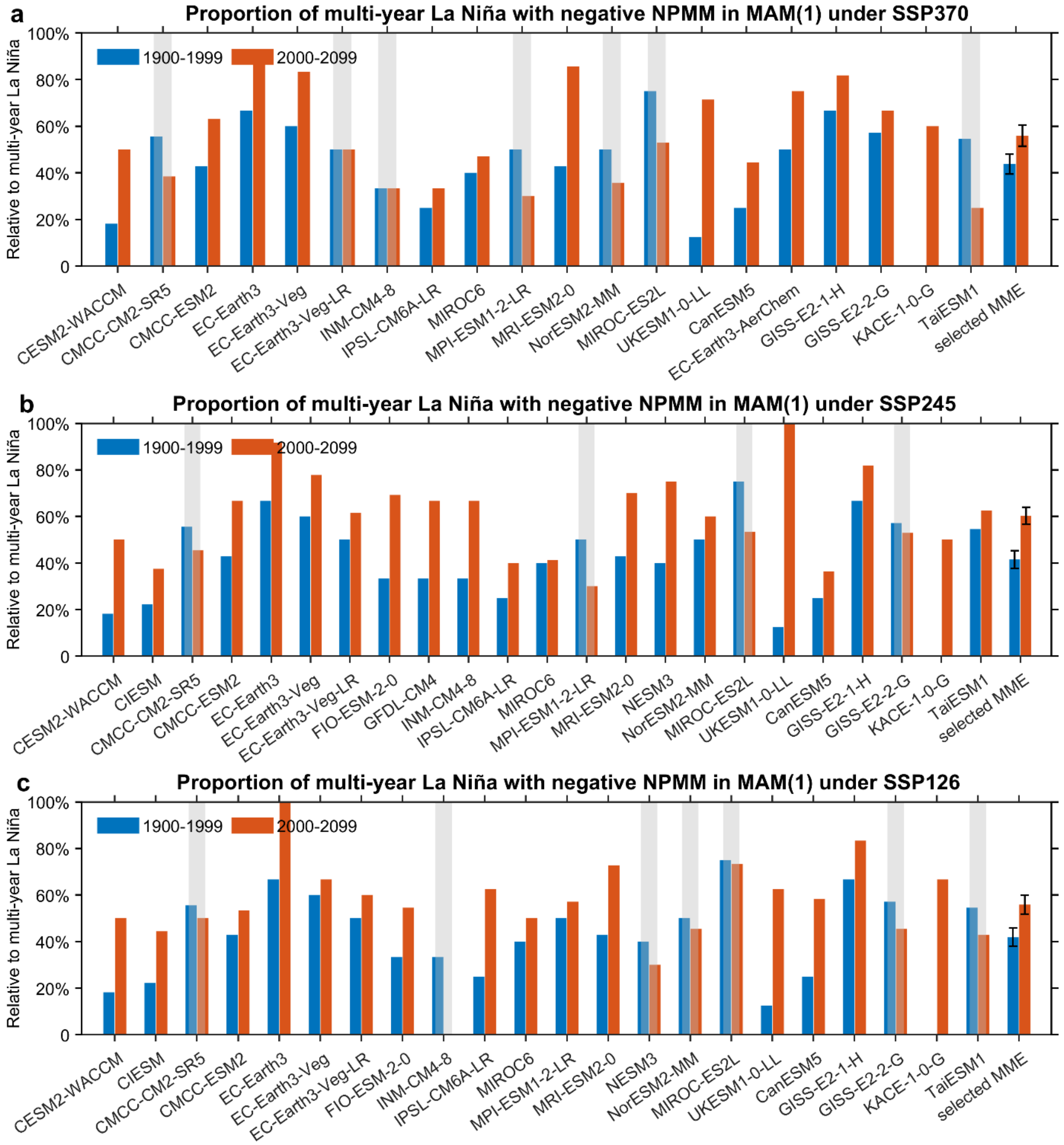

Supplementary Fig. 6 | Projected increase in co-occurrences of multi-year La Niña with negative NPMM-like events in MAM(1) in other emission scenarios. As in Extended Data Fig. 9a, but for a comparison of proportion (in percentage) of multi-year La Niña with negative NPMM-like events in MAM(1) relative to all multi-year La Niña events, over 1900-1999 (blue bars) and 2000-2099 (red bars) in the selected models under (**a**) SSP370, (**b**) SSP245, and (**c**) SSP 126 scenarios. A negative NPMM-like event is defined as when the NPMM index (normalized SST anomalies in 15°N-25°N, 150°W-120°E) is less than -0.5 s.d. in MAM. Models that simulate a decrease are greyed out. Error bars on the multi-model mean are calculated as 1.0 s.d. of 10,000 inter-realizations of a Bootstrap method.

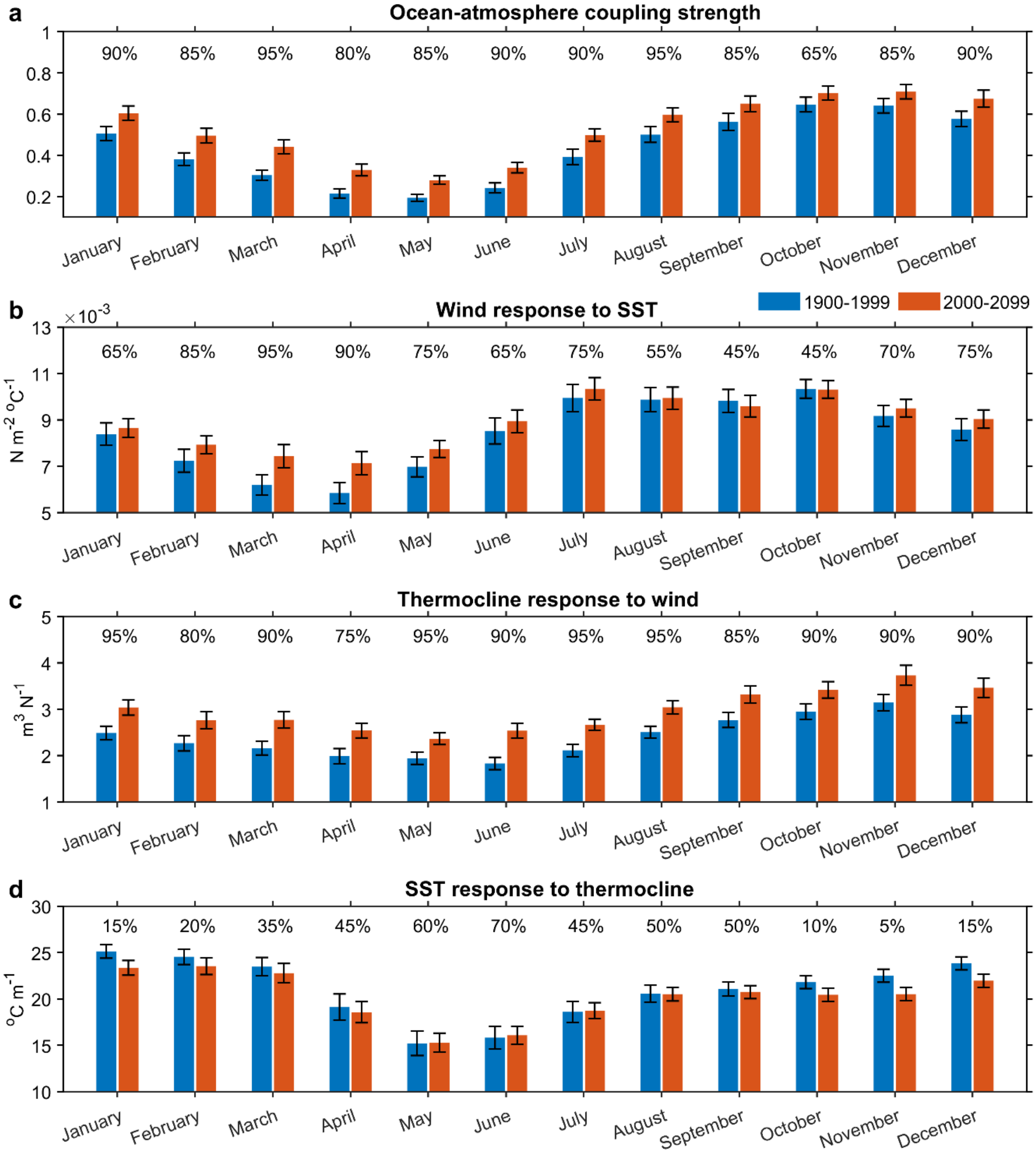

Supplementary Fig. 7 | Projected increase in ocean-atmosphere coupling strength in the equatorial Pacific. a, Comparison of ocean-atmosphere coupling strength in each calendar month over 1900-1999 (blue bars) and 2000-2099 (red bars). The coupling strength is defined as a product of three ocean-atmosphere coupled feedbacks. These feedbacks are: (**b**) central equatorial Pacific (5°S-5°N, 150°E-150°W) zonal wind response (regression coefficient; N m^{-2} $^{o}C^{-1}$) to Niño3.4 SST anomalies, (**c**) central-eastern equatorial Pacific (5°S-5°N, 170°W-90°W) sea surface height (SSH) response (regression coefficient; m^3 N^{-1}) to zonal wind anomalies, and (**d**) Niño3.4 SST response (regression coefficient; °C m^{-1}) to SSH anomalies. Shown are the multi-model ensemble mean results based on the selected models. Error bars are calculated as 1.0 s.d. of 10,000 inter-realizations of a Bootstrap method. The percentage of models that simulate an increase are labeled on the top of respective bars.

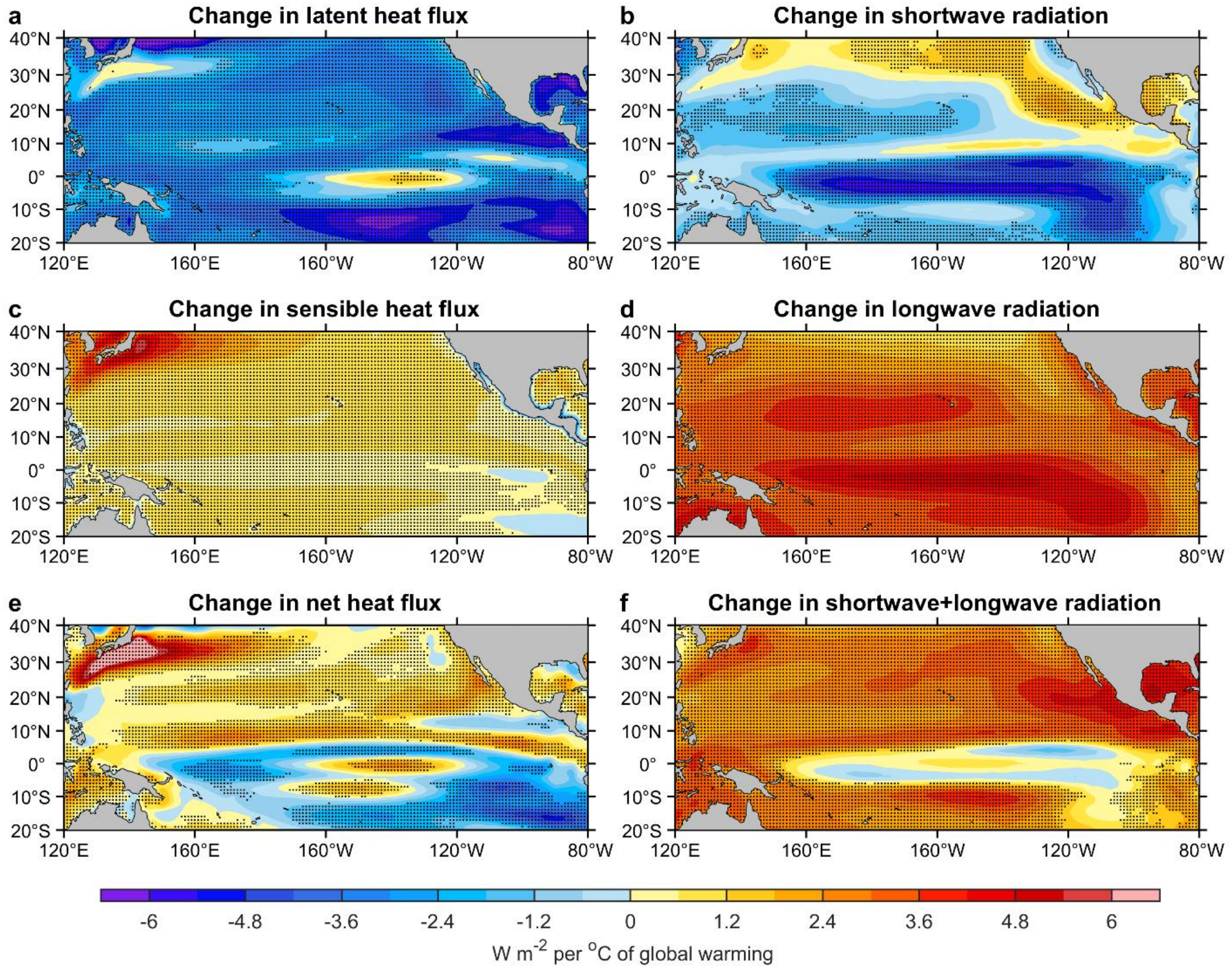

Supplementary Fig. 8 | Projected mean-state change in surface heat flux fields. a, Multi-model mean change of grid-point surface latent heat flux (positive downward into the ocean; W m^{-2}) between 1900-1999 and 2000-2099, scaled by the increase in global mean SST of each model. **b-f**, As in **a**, but for (**b**) shortwave radiation, (**c**) sensible heat flux, (**d**) longwave radiation, (**e**) net heat flux and (**f**) the sum of shortwave and longwave radiation. Stippling indicates that more than 80% of models show a same-signed change. The subtropical northeastern warming maximum is contributed by a direct heating from changes in shortwave and longwave radiation.

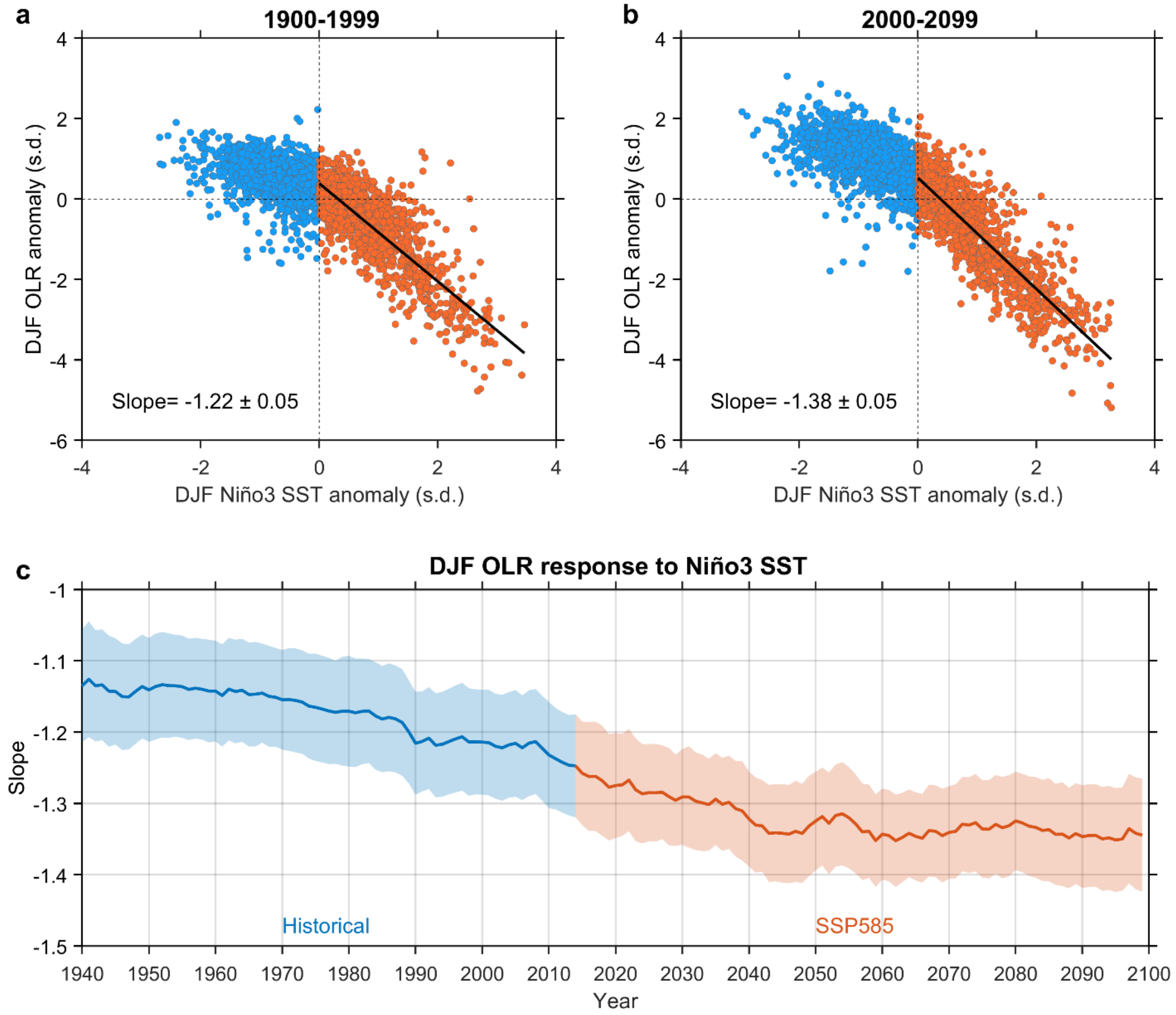

Supplementary Fig. 9 | Increased sensitivity of atmospheric response to equatorial Pacific SST heating. a, Relationship between normalized Niño3 SST anomalies (s.d.) and normalized outgoing longwave radiation (OLR; s.d.) anomalies in the central-eastern equatorial Pacific (5°S-5°N, 160°E-120°W) during DJF for 1900-1999 in the selected models. Also shown is the sensitivity measured by slope (black solid line) with 95% confidence intervals using positive Niño3 SST anomalies only (brown dots). **b,** As in **a,** but for 2000-2099. **c,** Evolution of the slope in a 60-year sliding window from historical periods to the 21st century under a high-emission scenario SSP585. Years on the x-axis denote the end year of the sliding window. Solid lines and shadings indicate multi-model mean and 1.0 standard deviation of a total of 10,000 inter-realizations based on a Bootstrap method, respectively. The convection response to the equatorial eastern Pacific SST anomalies increases but plateaus at approximately 2050.

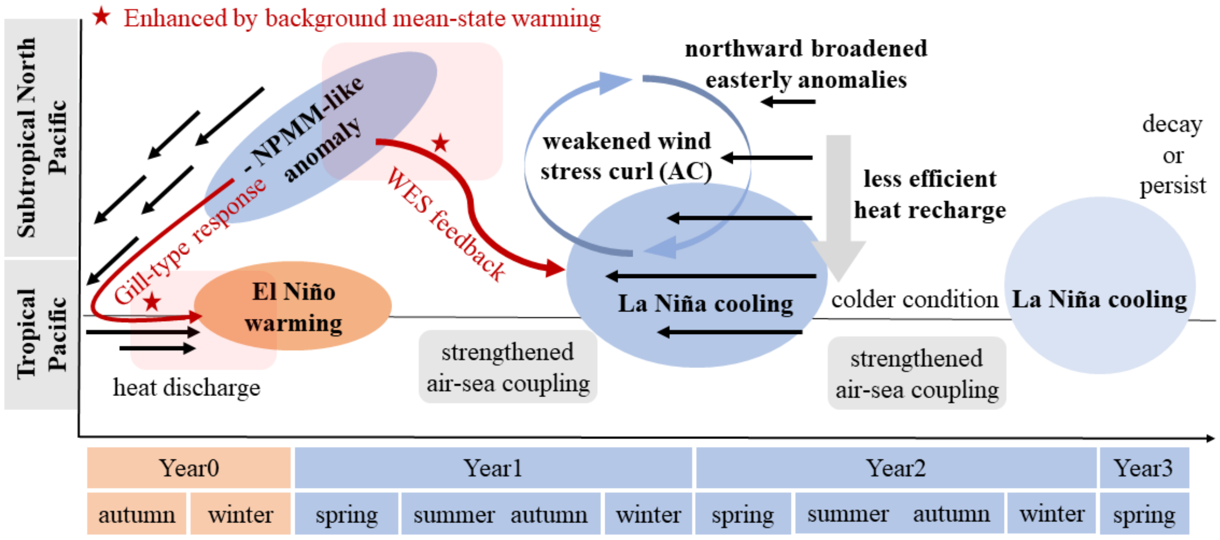

Supplementary Fig. 10 | Schematic depicting the mechanism for increased frequency of consecutive La Niña events under greenhouse warming. In addition to heat discharge in the tropical Pacific, an El Niño warm anomaly in the equatorial eastern Pacific induces a negative NPMM-like SST and wind anomaly (black arrows) in the subtropical northeastern Pacific at least partly through a Gill-type atmospheric response (red arrow). The negative NPMM-like pattern that features increased extratropical trade winds grows in boreal spring-summer under the WES feedback, favoring development of a meridionally broad La Niña in the following winter. The meridionally wide pattern of SST and easterly wind anomalies of the La Niña is accompanied by a weak anticyclonic (AC) wind stress curl at more-extratropical latitudes, slowing the heat recharge of the equatorial Pacific. Therefore, the cold SST anomalies are able to persist through the decaying phase of the first-year La Niña into spring, and with a seasonal strengthened air-sea coupling in summer and autumn, the remaining cold SST anomalies re-intensify and develop into another La Niña event in the next winter. Under transient greenhouse warming, a faster mean-state warming in the subtropical northeastern Pacific favors a northward broadened easterly wind anomaly by enhancing the WES feedback more to the north, boosting a stronger and more sensitive negative NPMM-like response to an equatorial eastern Pacific warm anomaly, which is further strengthened by a faster mean-sate warming in the equatorial eastern Pacific. The consequence of the northward broadened easterlies is a slower heat recharge in the upper equatorial Pacific (indicated by thick gray arrow) because of a weakened negative wind stress curl (blue circular arrows), facilitating the cold anomalies of the first-year La Niña to persist into the second year.

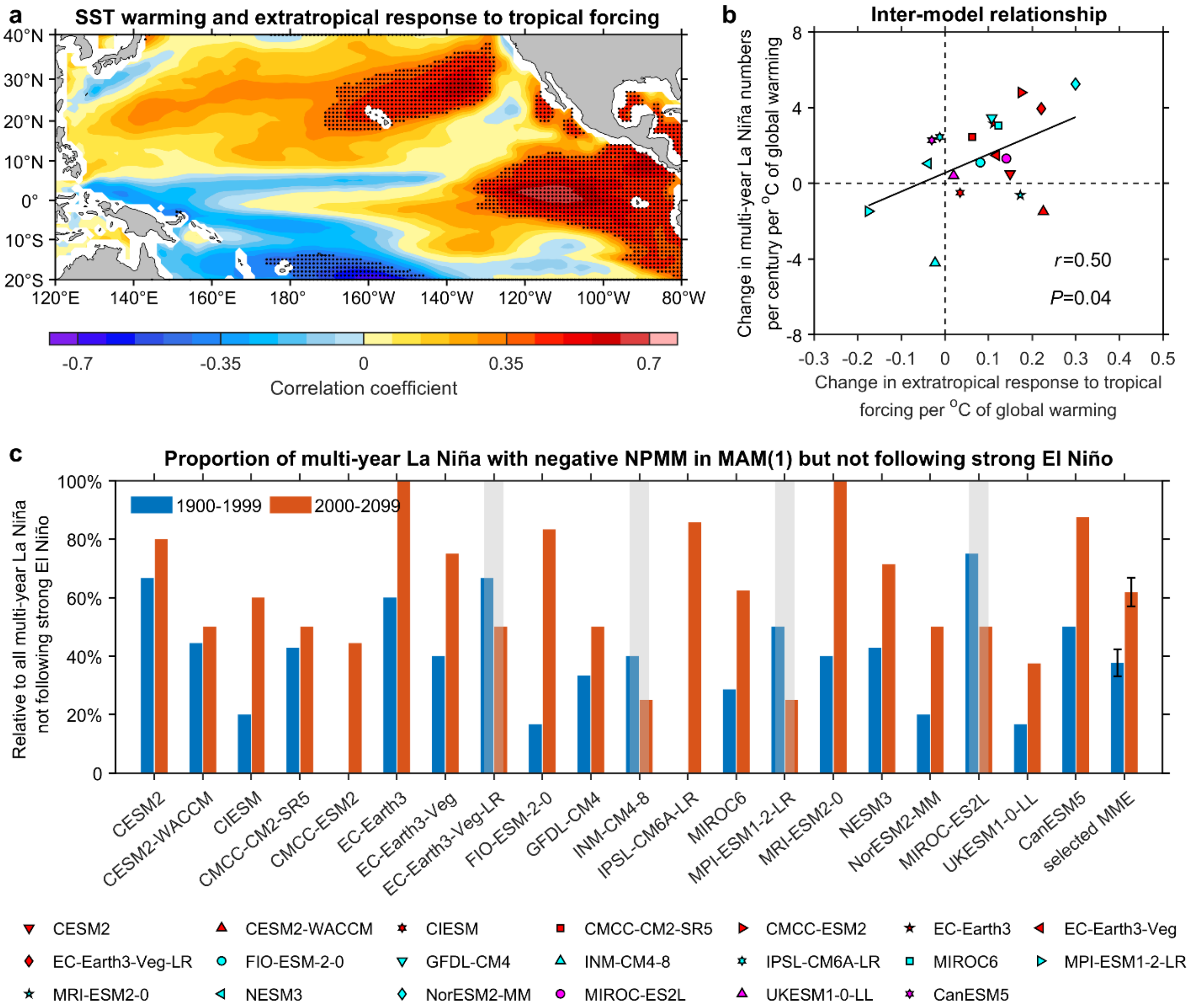

Supplementary Fig. 11 | Strengthened extratropical response to tropical forcing without the influence of strong El Niño. a, Inter-model correlation between changes in grid-point mean SST with change in the strength of extratropical response to tropical forcing after excluding strong El Niño years in both the SST and SLP expansion coefficients. Changes are scaled by the increase in global mean SST of each model. Stippling indicates statistical significance above the 90% level based on a two-tailed Student's *t*-test. **b**, Inter-model relationship between changes in multi-year La Niña numbers and in strength of the extratropical response to tropical forcing after excluding strong El Niño years in both the SST and SLP expansion coefficients. A linear fit (solid black line) is displayed together with correlation coefficient R and corresponding p value. **c,** Comparison of proportion (in percentage) of multi-year La Niña events that co-occur with negative NPMM-like events in MAM(1) but not follow strong El Niño relative to all multi-year La Niña events that are not preceded by strong El Niño, over 1900-1999 (blue bars) and 2000-2099 (red bars) in the selected models. A negative NPMM-like event is defined as when the NPMM index (normalized SST anomalies in 15°N-25°N, 150°W-120°E) is less than -0.5 s.d. in MAM. Error bars on the multi-model mean are calculated as 1.0 s.d. of 10,000 inter-realizations of a Bootstrap method. Like the increased response to strong El Niño in Fig. 4, a stronger response of extratropical anomalies to other El Niño events occurs in the 2000-2099 than in the 1900-1999 period.

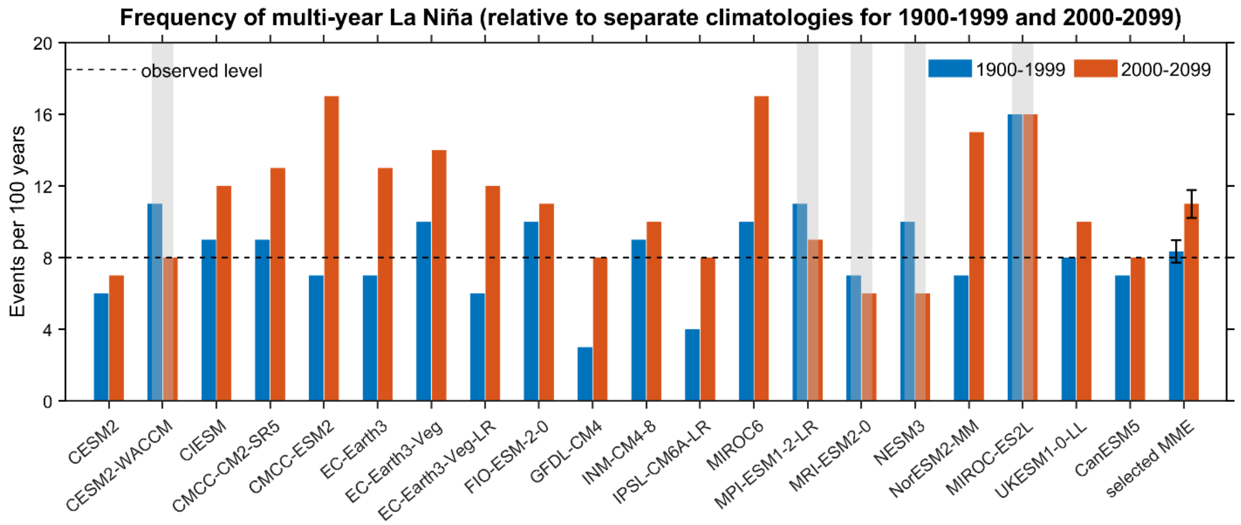

Supplementary Fig. 12 | Sensitivity of identification of multi-year La Niña events to climatology used for computing SST anomalies. Comparison of multi-year La Niña frequency (events per 100 years) over 1900-1999 (blue bars) and 2000-2099 (red bars) in the selected models under SSP585. Here the SST anomalies are calculated separately for 1900-1999 and 2000-2099, with reference to the monthly climatology of 1900-1999 and 2000-2099, respectively, and detrended. Models that do not simulate an increase are greyed out. Error bars on the multi-model mean are calculated as 1.0 standard deviation of 10,000 inter-realizations of a Bootstrap method. The horizontal dashed line indicates observation.

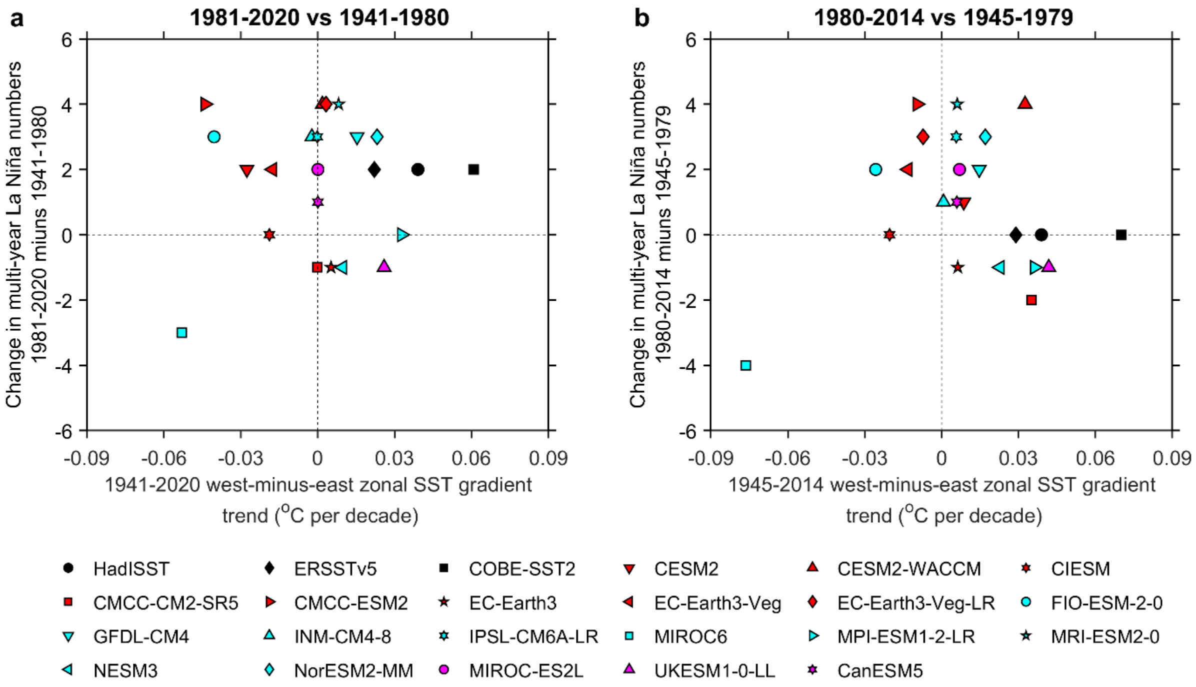

Supplementary Fig. 13 | Multi-year La Niña frequency and mean equatorial Pacific zonal SST gradient during recent decades. a, Relationship between change (1981-2020 minus 1941-1980) in multi-year La Niña numbers and trend in mean equatorial Pacific zonal SST gradient (°C per decade) during 1941-2020. Shown are results from the selected CMIP6 models and three observational reanalysis datasets (black symbols). The zonal SST gradient is defined as the SST difference between the western Pacific (150°E-180°, 5°N-5°S) and the central-eastern Pacific (150°W-120°W, 5°N-5°S), so that a positive trend in the gradient represents a faster warming in the western than eastern equatorial Pacific. **b,** As in **a,** but for the period of 1945-2014.

Supplementary Table 1 | Details of models. The CMIP6 models used in this study. Where there are multiple realizations, we use the first experiment that is available as indicated in the second column. The skewness of Niño3.4 SST anomalies in 1900-1999 are shown in the third column (positive in bold; observed value is 0.31 in three reanalysis datasets). Models with a positive skewness are selected and highlighted in bold. Note that not all models have experiments for each of the four warming scenarios.

Model name	Realization	Niño3.4 skewness	Emission Scenarios	Selected or not
ACCESS-CM2	r1i1p1f1	-0.231	SSP585, SSP370, SSP245, SSP126	N
ACCESS-ESM1-5	r1i1p1f1	-0.450	SSP585, SSP370, SSP245, SSP126	N
BCC-CSM2-MR	r1i1p1f1	-0.462	SSP585, SSP370, SSP245, SSP126	N
CAMS-CSM1-0	r1i1p1f1	-0.126	SSP585, SSP370, SSP245, SSP126	N
**CESM2**	r1i1p1f1	**0.044**	SSP585	**Y**
**CESM2-WACCM**	r1i1p1f1	**0.293**	SSP585, SSP370, SSP245, SSP126	**Y**
**CIESM**	r1i1p1f1	**0.400**	SSP585, SSP245, SSP126	**Y**
**CMCC-CM2-SR5**	r1i1p1f1	**0.549**	SSP585, SSP370, SSP245, SSP126	**Y**
**CMCC-ESM2**	r1i1p1f1	**0.531**	SSP585, SSP370, SSP245, SSP126	**Y**
**EC-Earth3**	r1i1p1f1	**0.353**	SSP585, SSP370, SSP245, SSP126	**Y**
**EC-Earth3-Veg**	r1i1p1f1	**0.501**	SSP585, SSP370, SSP245, SSP126	**Y**
EC-Earth3-CC	r1i1p1f1	-0.140	SSP585, SSP245	N
**EC-Earth3-Veg-LR**	r1i1p1f1	**0.254**	SSP585, SSP370, SSP245, SSP126	**Y**
FGOALS-f3-L	r1i1p1f1	-0.141	SSP585, SSP370, SSP245, SSP126	N
FGOALS-g3	r1i1p1f1	-0.398	SSP585, SSP370, SSP245, SSP126	N
**FIO-ESM-2-0**	r1i1p1f1	**0.259**	SSP585, SSP245, SSP126	**Y**
**GFDL-CM4**	r1i1p1f1	**0.064**	SSP585, SSP245	**Y**
GFDL-ESM4	r1i1p1f1	-0.014	SSP585, SSP370, SSP245, SSP126	N
IITM-ESM	r1i1p1f1	-0.354	SSP585, SSP370, SSP245, SSP126	N
**INM-CM4-8**	r1i1p1f1	**0.138**	SSP585, SSP370, SSP245, SSP126	**Y**
INM-CM5-0	r1i1p1f1	-0.065	SSP585, SSP370, SSP245, SSP126	N
**IPSL-CM6A-LR**	r1i1p1f1	**0.178**	SSP585, SSP370, SSP245, SSP126	**Y**
KIOST-ESM	r1i1p1f1	-0.160	SSP585, SSP245, SSP126	N
**MIROC6**	r1i1p1f1	**0.453**	SSP585, SSP370, SSP245, SSP126	**Y**
MPI-ESM1-2-HR	r1i1p1f1	-0.215	SSP585, SSP370, SSP245, SSP126	N
**MPI-ESM1-2-LR**	r1i1p1f1	**0.228**	SSP585, SSP370, SSP245, SSP126	**Y**
**MRI-ESM2-0**	r1i1p1f1	**0.217**	SSP585, SSP370, SSP245, SSP126	**Y**
**NESM3**	r1i1p1f1	**0.279**	SSP585, SSP245, SSP126	**Y**
NorESM2-LM	r1i1p1f1	-0.081	SSP585, SSP370, SSP245, SSP126	N
**NorESM2-MM**	r1i1p1f1	**0.046**	SSP585, SSP370, SSP245, SSP126	**Y**
CNRM-CM6-1	r1i1p1f2	-0.351	SSP585, SSP370, SSP245, SSP126	N
CNRM-CM6-1-HR	r1i1p1f2	-0.231	SSP585, SSP370, SSP245, SSP126	N
CNRM-ESM2-1	r1i1p1f2	-0.525	SSP585, SSP370, SSP245, SSP126	N
GISS-E2-1-G	r1i1p1f2	-0.120	SSP585, SSP370, SSP245, SSP126	N
**MIROC-ES2L**	r1i1p1f2	**0.350**	SSP585, SSP370, SSP245, SSP126	**Y**
**UKESM1-0-LL**	r1i1p1f2	**0.099**	SSP585, SSP370, SSP245, SSP126	**Y**
**CanESM5**	r1i1p2f1	**0.043**	SSP585, SSP370, SSP245, SSP126	**Y**
CAS-ESM2-0	r1i1p1f1	-0.087	SSP370, SSP245, SSP126	N
CESM2-FV2	r1i2p2f1	-0.092	SSP370	N
**EC-Earth3-AerChem**	r1i1p1f1	**0.27**	SSP370	**Y**

**GISS-E2-1-H**	r1i1p1f2	**0.16**	SSP370, SSP245, SSP126	**Y**
**GISS-E2-2-G**	r1i1p3f1	**0.28**	SSP370, SSP245, SSP126	**Y**
HadGEM3-GC31-LL	r1i1p1f3	-0.24	SSP245, SSP126	N
HadGEM3-GC31-MM	r1i1p1f3	-0.18	SSP126	N
IPSL-CM5A2-INCA	r1i1p1f1	-0.13	SSP370, SSP126	N
**KACE-1-0-G**	r1i1p1f1	**0.06**	SSP370, SSP245, SSP126	**Y**
**TaiESM1**	r1i1p1f1	**0.55**	SSP370, SSP245, SSP126	**Y**
UKESM1-1-LL	r1i1p1f2	-0.11	SSP370, SSP126	N
